# A resonant single frequency molecular detector with high sensitivity and selectivity for gas mixtures

**DOI:** 10.1038/s41598-020-58473-x

**Published:** 2020-01-30

**Authors:** Zorica Branković, Yuri Rostovtsev

**Affiliations:** 1 Center of Excellence for Green Technologies, Institute for Multidisciplinary Research, University of Belgrade, Kneza Viseslava 1a, 11030 Belgrade, Serbia; 2 Center for Nonlinear Sciences and Department of Physics, University of North Texas, Denton, TX 76203 USA

**Keywords:** Atomic and molecular physics, Atomic and molecular interactions with photons, Physics, Quantum physics, Quantum mechanics

## Abstract

Air quality control is an important task in prevention of human exposure to toxic and harmful gases and requires reliable gas sensors. During last decades many gas sensing mechanisms, based on different physical or chemical interactions with sensitive materials, have been developed, but the problem of precise analysis of gas mixtures still remains. The problem can be solved by introducing new sensing mechanism based on an adiabatically changing electric field interacting with the rotational structure of the molecules with dipole moments. We have theoretically demonstrated a single low frequency gas detector that can be used for sensing of gas mixtures with high selectivity, accuracy, and sensitivity. The enhancement of the population difference between corresponding molecular levels and reached the theoretical maximum of absorption have been shown.

## Introduction

The processes of dangerous pollution and the presence of toxic and harmful gases are important to understand in order to prevent their emissions. Prevention of dangerous gas emission starts from air quality control using gas sensing devices. Gas sensing refers to converting gas sensitive physical or chemical changes into signals that can be measured. Many techniques have been used for molecular detection^1,2^, e.g. optical^3,4^, calorimetric^5^, acoustic^6^, as well as methods based on variation in electrical properties, such as metal oxide semiconductor sensors^7^. Recent research efforts have resulted in a significant increase in sensitivity, reaching ppb range^8,9^, but selectivity and cross-sensing remain important research challenges. It would be of great importance to have a sensor that can efficiently analyze the multi-gas mixtures with high selectivity^10^. Such a gas sensor can be used for a huge range of applications – stretching from technology, sciences, control of environment, biology and medicine^11^. The scientific community is on relentless search for discover new techniques in order to overcome limitations of current approaches. For example, can a resonant frequency to detect a gas mixture of molecules be chosen by a researcher? Can detection signals be enhanced to reach maximum of molecular absorption rates?

It is well-know that each molecule has unique spectral transitions, which requires the use of corresponding resonant radiation with relatively high frequency. Optical radiation is resonant to electronic molecular transitions. Infrared and Raman spectroscopy takes advantage of using vibrational structure of molecules^12,13^, and the rotational transitions have frequencies in the so-called far-infrared region. Usually, for optical and IR transition, the conditions $kT\ll \hslash {\omega }_{opt}$ and $kT\simeq \hslash {\omega }_{vib}$ are satisfied and the population difference is close to maximal. For the rotational transitions, the population difference is really small because of $kT\gg \hslash {\omega }_{rot}$.

In this Report, we demonstrate that the researcher can choose a single low frequency electric field resonant to molecular transition with selectivity for multiple gas sensing with high accuracy and sensitivity. Using proper tailoring of the adiabatically changing electric field allows researchers to resonantly enhance the sensor signal. More, the tailoring increases the population difference between corresponding molecular levels and reaches the theoretical maximum of absorption.

## Results and Discussion

**Figure 1:**
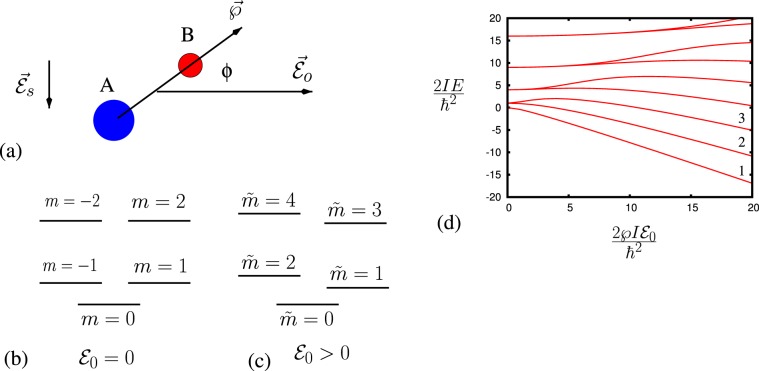
(**a**) Molecular rotator with electric dipole $\overrightarrow{\wp }$, where $\varphi$ is the angle of rotation with respect to the electric field ${\overrightarrow{{\mathcal{E}}}}_{0}$, and the ac electric field ${\overrightarrow{{\mathcal{E}}}}_{s}$ orthogonal to the dc field that is tuned to the resonance with the molecules transitions; (**b**) the energy structure of the molecular rotator with no electric field (${{\mathcal{E}}}_{0}=0$); (**c**) the energy structure of the molecular rotator with electric field (${{\mathcal{E}}}_{0} > 0$); (**d**) Molecular energy $2I{E}_{\tilde{m}}/{\hslash }^{2}$ dependence on $2I\wp {{\mathcal{E}}}_{0}/{\hslash }^{2}$. The numbers 1, 2, and 3 correspond to the states with $\tilde{m}=0$, $\tilde{m}=1$, and $\tilde{m}=2$ correspondingly.

We consider molecules with electric dipole moments, which allows for transitions between rotational levels. We can view symmetric molecules as simple rotators (see Fig. 1(a)) to simplify our consideration. The Hamiltonian of the molecule with dipole $\overrightarrow{\wp }$ in the dc electric field ${\overrightarrow{{\mathcal{E}}}}_{0}$ is given by

1 $$\hat{H}=\frac{{\hat{L}}_{z}^{2}}{2I}-\overrightarrow{\wp }\cdot {\overrightarrow{{\mathcal{E}}}}_{0},$$

where ${\hat{L}}_{z}$ is the *z* component of the operator of angular momentum, *I* is the molecular moment of inertia. Without electric field, the molecular energy spectrum is ${E}_{m}=\frac{{\hslash }^{2}}{2{I}}{{m}}^{2}$, the energies are degenerate for ±*m*, ${E}_{m}={E}_{-m}$.

Applying a dc electric field modifies the rotational energy levels of molecules (see Fig. 1(b,c)). The states $|\tilde{m}\rangle$ in the dc electric field with the rotational states $|m\rangle$ (without electric field) are related as

$$|\tilde{0}\rangle \to |m=0\rangle ,\,{\rm{for}}\,m > 0,\,|\tilde{m}=2|m|-1,2|m|\rangle \to |\pm |m|\rangle $$

The energy modification can be easily understood by naively considering aligned molecules in the dc electric field^14,15^. Aligned molecules can be viewed as simple pendulums. For levels with low energy excitation, they have the energy structure of a simple harmonic oscillator (see Fig. 1(a)). For higher excitation levels, the molecules retain rotational structure.

The dependence of the molecular energy $\frac{2IE}{{\hslash }^{2}}$ on the $\frac{2I\wp {{\mathcal{E}}}_{0}}{{\hslash }^{2}}$ is shown in Fig. 1(d). It shows that for the larger fields $\wp {{\mathcal{E}}}_{0}\gg \frac{{\hslash }^{2}}{I}$, the electric dipole under the presence of the electric field looks like a simple harmonic oscillator, with the Hamiltonian given by

2 $$\hat{H}=-\,\frac{{\hslash }^{2}}{2I}\frac{{\partial }^{2}}{\partial {\varphi }^{2}}+\frac{I{\omega }_{E}^{2}}{2}{\varphi }^{2}-\wp {{\mathcal{E}}}_{0},$$

where ${\omega }_{E}^{2}=\frac{\wp {{\mathcal{E}}}_{0}}{I}$. As can be seen in Fig. 1(b), the ground and lower states have energies

3 $${E}_{\tilde{m}}\simeq -\,\wp {{\mathcal{E}}}_{0}+\hslash {\omega }_{E}(\tilde{m}+1/2).$$

Meanwhile, for larger $\tilde{m}$ such that $\frac{{\hslash }^{2}}{2I}{\tilde{m}}^{2}\gg \wp {{\mathcal{E}}}_{0}$, the fast molecular rotation completely averages out the effect of the dc electric field, and the molecular energy is ${E}_{\tilde{m}}\simeq \frac{{\hslash }^{2}}{2I}{\tilde{m}}^{2}$.

The modification of the energy structure also results in the redistribution of the population in the rotational levels. Populations on the molecular rotational levels can be estimated as

4 $${N}_{\tilde{m}}=\frac{{e}^{-\frac{{E}_{\tilde{m}}}{kT}}}{Z}{N}_{0},\,{\rm{where}}\,Z=\mathop{\sum }\limits_{\tilde{m}=0}^{\infty }\,{e}^{-\frac{{E}_{\tilde{m}}}{kT}}$$

where $k$ is the Boltzmann constant, $T$ is the room temperature, and ${N}_{0}$ is the molecular gas density.

**Figure 2:**
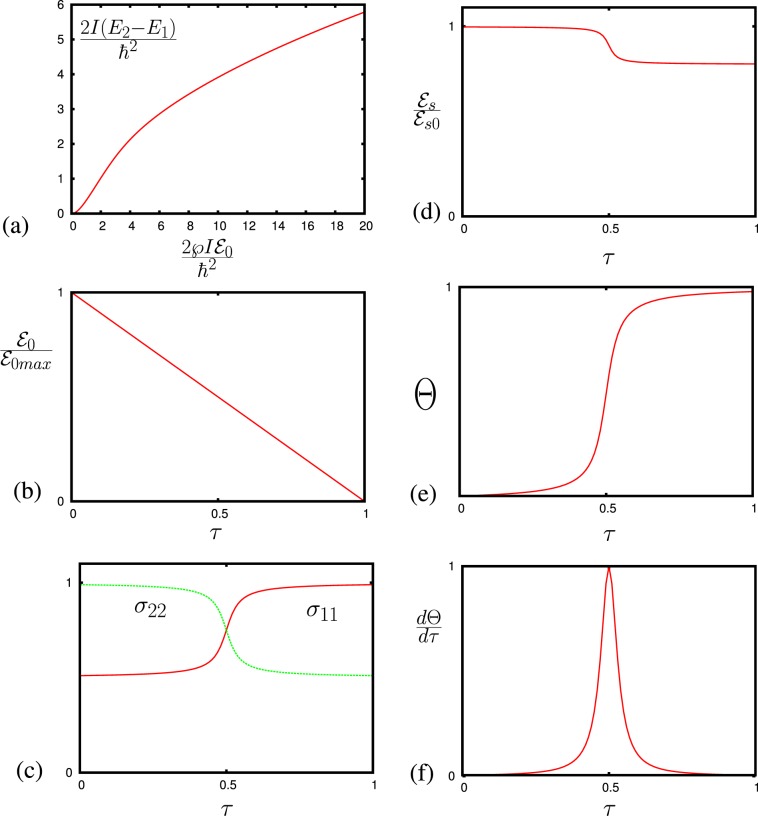
(**a**) Dependence of the molecular transition $2I({E}_{\tilde{2}}-{E}_{\tilde{1}})/{\hslash }^{2}$ as a function of $2I\wp {{\mathcal{E}}}_{0}/{\hslash }^{2}$. (**b**) Adiabatic change of the electric field ${{\mathcal{E}}}_{0}/{{\mathcal{E}}}_{0max}$ vs time $\tau =\alpha t$; (**c**) Dependence of the populations ${\sigma }_{11}$ and ${\sigma }_{22}$ (arbitrary unites) in levels $\tilde{m}=1$ and $\tilde{m}=2$ on time $\tau$. (**d**) Dependence of $|{{\mathcal{E}}}_{s}/{{\mathcal{E}}}_{s0}|$ on time. (**e**) Dependence of phase $\Theta$ (arbitrary unites) of electric field ${{\mathcal{E}}}_{s}/|{{\mathcal{E}}}_{s0}|$ on time $\tau$. (**f**) Dependence of $\frac{d\Theta }{d\tau }$ (arbitrary unites) on time $\tau$.

We can completely align the molecules along the electric field by applying sufficiently strong electric field ${{\mathcal{E}}}_{0}$. Then, we can adiabatically change the electric field (see in Fig. 2(d)) so that the molecules would follow the field (${{\mathcal{E}}}_{0}={{\mathcal{E}}}_{0,max}(1-\alpha t)$ where *α* is the rate of field change and it should be smaller than the splitting between levels, $\alpha \ll ({E}_{2}-{E}_{1})/\hslash$), and the molecules populated at the lowest level will end up in the lowest rotational energy state (the rate of field change should be faster than the relaxation rate). For the case of higher temperature or not strong enough electric field, the population will be distributed among lower rotational levels (not only at the lowest rotational level). Thus, the population difference between levels $\tilde{m}=1$ and $\tilde{m}=2$ (see in Fig. 1(d)) is given by

5 $${N}_{2}-{N}_{1}=\frac{{E}_{2}-{E}_{1}}{kTZ}{N}_{0}.$$

Notice that this is similar to the magnetic cooling with the adiabatically changing magnetic field^16–18^.

The first excited rotational state degenerated by the direction of rotation without dc electric field (see Fig. 1(b)) is split in the dc electric field due to the Stark effect (see Fig. 1(c)). The splitting depends on the magnitude of the electric field, and changes from zero (no electric field) to some energy that is determined by the frequency of the oscillation of the pendulum of the aligned molecule $({\omega }_{E}=\sqrt{\frac{\wp {{\mathcal{E}}}_{0}}{I}})$.

This energy splitting depends on ${{\mathcal{E}}}_{0}$

$$\frac{2I({E}_{2}-{E}_{1})}{{\hslash }^{2}}=F\left(\frac{2\wp I{{\mathcal{E}}}_{0}}{{\hslash }^{2}}\right)$$

and is shown in Fig. 2(a). Splitting can be tuned to any frequency ${\omega }_{0}=\frac{({E}_{2}-{E}_{1})}{\hslash }$, and the allowed dipole transition can be used for spectroscopy. The strong (dc or slowly varying) electric field controls both the population and the splitting between levels, and the ac electric field orthogonal to the dc field is tuned to the resonance with the molecules transitions (see Fig. 2(b)). The ac field ${{\mathcal{E}}}_{s}$ is created by the high fineness resonance circuit. The equation for the Rabi frequency ${\Omega }_{s}=\wp {{\mathcal{E}}}_{s}/\hslash$ is given by

6 $${\dot{\Omega }}_{s}=-\,i({\omega }_{0}-{\omega }_{s}){\Omega }_{s}+i{\Omega }_{a}^{2}{\sigma }_{ab}$$

where ${\omega }_{s}$ is the frequency of the resonance circuit, ${\Omega }_{a}^{2}=\frac{2\pi {\omega }_{0}{\wp }^{2}N}{\hslash }$ is the cooperative frequency, and ${\sigma }_{12}$ is the molecular coherence between the molecular states involved, and ${\sigma }_{12}$ is the element of the density matrix $\sigma$ that satisfies the Liouville equation

$$i\hslash \dot{\sigma }=[\hat{H},\sigma ].$$

The resonance depends on the dc electric field applied and the molecular parameters such as rotational constants and the magnitude of the molecular dipole moment. This opens an interesting opportunity for molecular detection at the chosen frequency. Different molecules exhibit their resonances at the frequency but at different magnitudes of the electric field. Knowing electric field and frequency allows the researcher to determine the molecules.

It is known that coherence effects play an essential role in atomic and molecular physics^19,20^. In particular, the pulse shaping in coherent Raman spectroscopy^21^ allows researchers to improve sensitivity.

**Figure 3:**
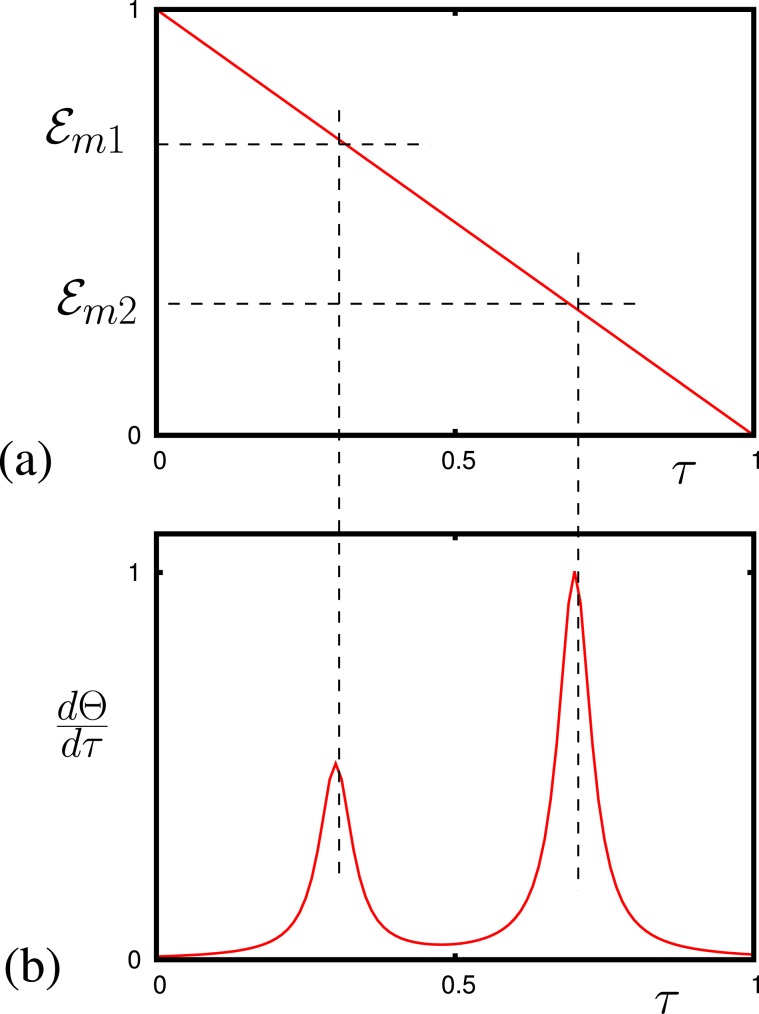
(**a**) Adiabatic change of the electric field ${{\mathcal{E}}}_{0}/{{\mathcal{E}}}_{0max}$ vs time $\tau$; dashed lines show the moment in time of the resonances with corresponding molecular transitions. (**c**) Dependence of $\frac{d\Theta }{d\tau }$ (arbitrary unites) for two molecular gases vs time.

The adiabatic change of the electric field provides us additional enhancement of the signal. Particularly, adiabatic tuning of the molecular levels across the resonance with an ac electric field, the complete adiabatic population transfer^20^ maximizes the amount of signal that the ac field can experience. The dynamics of population in the states is shown in Fig. 2(c). The ac field ${{\mathcal{E}}}_{s}=|{{\mathcal{E}}}_{s}|\,\exp (i\Theta )$ is absorbed because of the population transfer as well as the phase ($\Theta$) change of the ac field (see in Fig. 2(d,e)). Both the amplitude and the phase can be used for detecting the molecules with this technique. The time derivative of the phase $(\tfrac{d\Theta }{d\tau })$ gives us a molecular resonance (see in Fig. 2(f)). Thus, in the case when we have more than one molecular gas, for each gas we have condition for the electric field ${{\mathcal{E}}}_{m\beta }$ to observe the resonance

$$\frac{2{I}_{\beta }{\omega }_{0}}{\hslash }=F\,\left(\frac{2{\wp }_{\beta }{I}_{\beta }{{\mathcal{E}}}_{m\beta }}{{\hslash }^{2}}\right),$$

where $\beta$ is the index for each gas in the gas mixture. The phase $(\tfrac{d\Theta }{d\tau })$ gives us several resonances corresponding to each of the molecular gases in the mixture (see in Fig. 3, $\beta =1,2$).

Based on the theoretical principles explained above, we can make estimation of the technique for simple molecules such as shown in Table 1. The noise for microwaves and RF is determined by the power of thermal noise. Because the energy of quanta is small, the shot-noise is not important, and the main contribution expected coming from the thermal Johnson-Nyquist noise^22^. For the condition $\hslash {\omega }_{0}\gg kT$, and using the Rayleigh-Jeans approximation, the noise is determined by ${P}_{noise}=kT\Delta \omega$, where Δ$\omega$ is the width of signal detection, in this case, the width of the microwave (or RF) mode of radiation. In our case, the width is smaller than the relaxation rate $\Delta \omega  < \gamma$ ($\gamma \simeq {10}^{7}\,{{\rm{s}}}^{-1}$). In Table 1, we show estimations of the dc electric field that is needed to make a resonance with the ac electric field ${\omega }_{0}=(2\pi )500\,{\rm{MHz}}$. We can see that for molecules with small electric dipole, the magnitude of the dc field should be bigger to establish the resonance, but the resonance can be made at smaller dc fields with lower ac frequencies (see Eq. (M8)). We can see that each molecule has the resonance with the same frequency at different dc fields (see Table 1), all fields are different for different molecules. The sensitivity can be found by detecting the change of the phase $\Theta$ of the microwave (or RF) radiation, $\delta \Theta$. The fundamental limits for the minimal detection phase are determined by the signal to noise ratio, namely,

$$\delta \Theta \simeq \frac{\delta {P}_{s}}{{P}_{s}}=\frac{{P}_{noise}}{{P}_{s}}.$$

**Table 1:** Estimations for the parameters of some molecules (Molecular parameters have been taken from https://cccbdb.nist.gov/ and references there).

Molecules	Dipole	Rotational Constant	dc Electric field	Minimum density
CO,	${\wp }_{CO}$ = 0.122 D,	*B* = 1.93 cm^−1^;	45 10^3^ V/cm,	2 10^10^ cm^−3^
HCN,	${\wp }_{HCN}$ = 2.98 D,	*B* = 1.47 cm^−1^,	1600 V/cm,	3 10^10^ cm^−3^
N_2_O,	${\wp }_{{N}_{2}O}$ = 0.17 D,	*B* = 0.42 cm^−1^;	15 10^3^ V/cm,	9 10^10^ cm^−3^
NO,	${\wp }_{NO}$ = 0.16 D,	*B* = 1.67 cm^−1^,	30 10^3^ V/cm,	2 10^10^ cm^−3^
SO,	${\wp }_{SO}$ = 1.55 D,	*B* = 0.72 cm^−1^,	2200 V/cm,	5 10^10^ cm^−3^
KBr,	${\wp }_{KBr}$ = 10.6 D,	*B* = 0.08 cm^−1^,	106 V/cm,	5 10^9^ cm^−3^
NO_2_,	${\wp }_{N{O}_{2}}$ = 0.316 D,	*A* = 8.0 cm^−1^,	8300 V/cm,	9 10^10^ cm^−3^
		*B* = 0.43 cm^−1^,		
		*C* = 0.41 cm^−1^,		
H_2_O,	${\wp }_{{H}_{2}O}$ = 1.85 D,	*A* = 27.88 cm^−1^,	6500 V/cm,	4 10^9^ cm^−3^
		*B* = 14.512 cm^−1^,		
		*C* = 9.29 cm^−1^,		
O_3_,	${\wp }_{{O}_{3}}$ = 0.53 D,	*A* = 3.550 cm^−1^,	4800 V/cm,	1 10^11^ cm^−3^
		*B* = 0.44 cm^−1^,		
		*C* = 0.39 cm^−1^,		
CCl_2_O,	${\wp }_{CCl2O}$ = 1.17 D,	*A* = 0.26 cm^−1^,	1080 V/cm,	4 10^11^ cm^−3^
		*B* = 0.16 cm^−1^,		
		*C* = 0.08 cm^−1^,		
NH_3_,	${\wp }_{N{H}_{3}}$ = 1.47 D,	*A* = 9.44 cm^−1^,	6600 V/cm,	6 10^9^ cm^−3^
		*B* = 9.44 cm^−1^,		
		*C* = 6.19 cm^−1^,		

The change of $\delta \Theta$ is related to the molecular density by Eq. (M20), so we can estimate the minimum detectable molecular density $\delta {N}_{min}=\frac{\hslash \alpha }{2\pi {\omega }_{0}{\wp }^{2}}\frac{{P}_{noise}}{{P}_{s}}$ (shown in Table 1). We can see that the molecules that have smaller rotational constants have slightly higher detection densities than the molecules that have higher rotational constants. We can roughly summarized that using $\wp \simeq 1D$ as a typical value, and $B\simeq 1\,{{\rm{cm}}}^{-1}$, we obtain $N\simeq 1\,{10}^{11}\,{{\rm{cm}}}^{-3}$. We make estimation for some molecules that can be polutants and need to be controlled in the atmosphere, but also we make our estimation for molecules like *KBr* that can be used for quantum information^23^, and the sensor can be used for detection of their states.

In this paper, we have shown that the resonances for different molecules occur at different magnitudes of the dc electric fields, and thus allows researchers to detect each molecule in the mixture of molecular gases. Furthermore, the technique can be used for molecular systems with complicated rotational structures and a large electric dipole. The combination of the rotational motion and dipole moment allows one to uniquely detect these molecular systems or molecular clusters. Potentially, the technique can be extended for nanomaterials, where stronger fields are obtainable due to surface plasmonic resonances. With more complex field configurations, the effect of molecular alignment may be enhanced.

Recent discussions of gas detection using metal oxide gas sensors (MOS) in literature^24–26^ may also benefit from this technique. Studies have indicated that the gas sensing process is strongly related to surface reactions, changing the impedance of the electric circuit. The MOS has important advantages because the sensors can operate at low frequency. Our proposed technique can be applied to the MOS where tailored low frequency resonances can provide important contributions and improve technique.

## Methods

### Dipole molecules in dc electric fields

We consider the gas of molecules that have a permanent electric dipole $\overrightarrow{\wp }$. For simplicity let us model such molecules as simple rotators with fixed axis. To align the molecules with the permanent electric dipoles along the electric field, the dc (or slow varying) electric field is applied. The Hamiltonian of the molecule with dipole $\overrightarrow{\wp }$ in the dc electric field ${\overrightarrow{{\mathcal{E}}}}_{0}$ is given by

M1 $$\hat{H}=\frac{{\hat{L}}_{z}^{2}}{2I}-\overrightarrow{\wp }\cdot {\overrightarrow{{\mathcal{E}}}}_{0}=\frac{{\hat{L}}_{z}^{2}}{2I}-\wp {{\mathcal{E}}}_{0}\,\cos \,\varphi ,$$

where ${\hat{L}}_{z}$ is the $z$ component of the operator of angular momentum, $I$ is the molecular moment of inertia, and $\varphi$ is the angle between the dipole moment of the rotator and the dc electric field.

Note here that without electric field (${{\mathcal{E}}}_{0}=0$), the molecular energy spectrum is ${E}_{m}=\frac{{\hslash }^{2}}{2I}{m}^{2}$, the energies are degenerate for $m$ and −*m*, ${E}_{m}={E}_{-m}$, and the wave function corresponding to that energy is $\langle \varphi |m\rangle =\frac{1}{\sqrt{2\pi }}\,\exp [im\varphi ]$. Applying the electric field changes the molecular energies (see Fig. 1). The dependence of the molecular energies $\frac{2IE}{{\hslash }^{2}}$ on the $\frac{2\wp {E}_{0}}{{\hslash }^{2}}$ is shown in Fig. 1(d). For larger fields $\wp {{\mathcal{E}}}_{0}\gg \frac{{\hslash }^{2}}{I}$, the electric dipole under the presence of the electric field looks like a simple harmonic oscillator, and this can be seen from the above Hamiltonian as well,

M2 $$\hat{H}=-\,\frac{{\hslash }^{2}}{2I}\frac{{\partial }^{2}}{\partial {\varphi }^{2}}+\frac{I{\omega }_{E}^{2}}{2}{\varphi }^{2}-\wp {{\mathcal{E}}}_{0},$$

where $${\omega }_{E}^{2}=\frac{\wp {E}_{0}}{I}$$.

As can be seen in Fig. 1, the ground state (level 0) and lower states have energies

M3 $$\begin{array}{rcl}{E}_{\tilde{m}} & \simeq  & \mathop{\underbrace{\frac{\hslash {\omega }_{E}}{2}(2\tilde{m}+1)}}\limits_{{\rm{a}}\,{\rm{simple}}\,{\rm{harmonic}}\,{\rm{oscillator}}}\\  &  & -\wp {{\mathcal{E}}}_{0}-\frac{{\hslash }^{2}}{48I}({(2\tilde{m}+1)}^{2}+\tilde{m}(\tilde{m}-1)+(\tilde{m}+1)(\tilde{m}+2)),\end{array}$$

and for larger $\tilde{m}$ such that $\frac{{\hslash }^{2}}{2I}{\tilde{m}}^{2}\gg -\,\wp {E}_{0}+\frac{\hslash {\omega }_{E}}{2}(2\tilde{m}+1)$ the energy spectrum is

$${E}_{\tilde{m}}=\frac{{\hslash }^{2}}{2I}{\tilde{m}}^{2}+\frac{I{\wp }^{2}{{\mathcal{E}}}_{0}^{2}}{4{\hslash }^{2}{\tilde{m}}^{2}}.$$

Adiabatic switching off the dc electric field leads the states $|\tilde{m}\rangle$ back to rotational states $|m\rangle$

$$|\tilde{0}\rangle \to |m=0\rangle ,\,{\rm{for}}\,m > 0,\,|\tilde{m}=2|m|-1,2|m|\rangle \to |\pm |m|\rangle $$

Let us note here that the molecules without permanent electric dipole can also behave similarly. Indeed, the Hamiltonian of the molecule with dipole $\overrightarrow{\wp }$ induced in the dc electric field ${\overrightarrow{{\mathcal{E}}}}_{0}$ as $\overrightarrow{\wp }=\hat{n}{\alpha }_{z}{{\mathcal{E}}}_{0}\,\cos \,\varphi -\hat{l}{\alpha }_{x}{{\mathcal{E}}}_{0}\,\sin \,\varphi$ ($\hat{n}$ and $\hat{l}$ are unit vectors along and perpendicular to the orientation of the molecule) is given by

M4 $$\hat{H}=\frac{{\hat{L}}_{z}^{2}}{2I}-\overrightarrow{\wp }\cdot {\overrightarrow{{\mathcal{E}}}}_{0}$$

A simple harmonic oscillator can be also obtained

$$\begin{array}{rcl}\hat{H} & = & \frac{{\hat{L}}_{z}^{2}}{2I}-{\alpha }_{z}{{\mathcal{E}}}_{0}^{2}\,{\cos }^{2}\,\varphi -{\alpha }_{x}{{\mathcal{E}}}_{0}^{2}\,{\sin }^{2}\,\varphi \\  & \simeq  & -\frac{{\hslash }^{2}}{2I}\frac{{\partial }^{2}}{\partial {\varphi }^{2}}+\frac{I{\omega }_{E}^{2}}{2}{\varphi }^{2}\end{array}$$

where ${\omega }_{H}^{2}=\frac{({\alpha }_{z}-{\alpha }_{x})}{I}{{\mathcal{E}}}_{0}^{2}$. And all results obtained in the paper can be applied for these molecules too.

Now, we use the electric field that has the orthogonal polarization to couple the state $|1\rangle$ and $|2\rangle$, the Hamiltonian is given by

M5 $$\hat{H}=-\,\frac{{\hslash }^{2}}{2I}\frac{{\partial }^{2}}{\partial {\varphi }^{2}}-\wp {{\mathcal{E}}}_{0}\,\cos \,\varphi -\wp {{\mathcal{E}}}_{s}\,\sin \,\varphi $$

where ${\overrightarrow{{\mathcal{E}}}}_{s}$ is the time dependent electric field perpendicular to ${\overrightarrow{{\mathcal{E}}}}_{0}$. In perturbation limit, $\frac{{\hslash }^{2}}{2I}\gg \wp {{\mathcal{E}}}_{0}$, introducing $B=\frac{\hslash }{2I}$, and using matrix elements

M6 $$\langle {m}_{1}|{e}^{i\varphi }|{m}_{2}\rangle ={\delta }_{{m}_{1}+1,{m}_{2}}\,{\rm{and}}\,\langle {m}_{1}|{e}^{-i\varphi }|{m}_{2}\rangle ={\delta }_{{m}_{1},{m}_{2}+1}$$

we obtain

M7 $$|\,+\,\rangle =\frac{\sqrt{\frac{1}{2}}|\,+\,1\rangle +\frac{\sqrt{2}\wp {{\mathcal{E}}}_{0}}{\hslash B}|0\rangle +\sqrt{\frac{1}{2}}|\,-\,1\rangle }{\sqrt{1+\frac{2{\wp }^{2}{{\mathcal{E}}}_{0}^{2}}{{\hslash }^{2}{B}^{2}}}}\,{\rm{and}}\,|\,-\,\rangle =\frac{|\,+\,1\rangle -|\,-\,1\rangle }{\sqrt{2}}$$

The transition frequency is given by

M8 $${\omega }_{21}=\frac{2{\wp }^{2}{{\mathcal{E}}}_{0}^{2}}{{\hslash }^{2}B}$$

and the matrix element of transition is given by

M9 $$\langle \,+\,|\,\sin \,\varphi |\,-\,\rangle =\frac{i\frac{\wp {{\mathcal{E}}}_{0}}{\hslash B}}{\sqrt{1+\frac{2{\wp }^{2}{{\mathcal{E}}}_{0}^{2}}{{\hslash }^{2}{B}^{2}}}}.$$

The room temperature should be taken into account. Populations on the molecular rotational levels can be estimated as

M10 $${N}_{\tilde{m}}=\frac{{e}^{-\frac{{E}_{\tilde{m}}}{kT}}}{Z}{N}_{0},\,{\rm{where}}\,Z=\mathop{\sum }\limits_{\tilde{m}=1}^{\infty }\,{e}^{-\frac{{E}_{\tilde{m}}}{kT}}$$

where $k$ is the Boltzman constant, $T$ is the absolute temperature, ${N}_{0}$ is the molecular gas density. Thus, the population difference between levels 2 and 3 is given by

M11 $${N}_{2}-{N}_{3}=\frac{{E}_{\tilde{3}}-{E}_{\tilde{2}}}{kTZ}{N}_{0}.$$

### Adiabatic manipulations of populations in rotational levels

The density matrix equations are given by

M12 $$\frac{\partial \sigma }{\partial t}=\frac{i}{\hslash }[\sigma ,H]-\hat{\Gamma }[\sigma ],$$

where $\hat{\Gamma }[\sigma ]$ is the relaxation matrix for all components of the density matrix *σ*. The set of density matrix equations is given by

M13 $${\dot{\sigma }}_{ab}=-\,({\Gamma }_{ab}+i\Delta ){\sigma }_{ab}+i{n}_{ab}{\Omega }_{s},$$

M14 $${\dot{\sigma }}_{bb}=i({\sigma }_{ba}{\Omega }_{s}-{\Omega }_{s}^{\ast }{\sigma }_{ab}),$$

M15 $${\dot{\sigma }}_{aa}=i({\sigma }_{ab}{\Omega }_{s}^{\ast }-{\Omega }_{s}{\sigma }_{ba}),$$

where ${n}_{ab}={\sigma }_{aa}-{\sigma }_{bb}$, $\Delta ={\omega }_{ab}({{\mathcal{E}}}_{0})-{\omega }_{0}$, and ${\omega }_{ab}({{\mathcal{E}}}_{0})=\frac{{E}_{a}({{\mathcal{E}}}_{0})-{E}_{b}({{\mathcal{E}}}_{0})}{\hslash }$ is the transition frequency (see Fig. 2(a) for $a=1$ and $b=2$ in the paper). The typical behavior of the populations ${\sigma }_{11}$ and ${\sigma }_{22}$ is shown in Fig. 2(c) of the paper.

The ${{\mathcal{E}}}_{s}$ is the electric field in the resonant contour that contains the capacitor and the coil with the resonant frequency ${\omega }_{0}$. The capacitor is filled with a molecular gas. The equation for the field ${{\mathcal{E}}}_{s}$ is given by

M16 $${\mathop{{\mathcal{E}}}\limits^{..}}_{s}=-\,{\omega }_{0}^{2}{{\mathcal{E}}}_{s}+4\pi {\omega }_{0}^{2}P$$

where the molecular polarization is given by $P=\wp N{\sigma }_{ab}$. Introducing the Rabi frequency ${\Omega }_{s}=\wp {{\mathcal{E}}}_{s}$/$\hslash$, then the slowly-varying amplitude equation is given by

M17 $${\dot{\Omega }}_{s}=-\,i({\omega }_{0}-{\omega }_{ab}){\Omega }_{s}+\frac{2\pi i{\omega }_{0}{\wp }^{2}N}{\hslash }{\sigma }_{ab},$$

where ${\Omega }_{a}^{2}=\frac{2\pi {\omega }_{0}{\wp }^{2}N}{\hslash }$ is the cooperative frequency. The typical behavior of the Rabi frequency ${\Omega }_{s}$ ($|{\Omega }_{s}|$ and $\Theta$) is shown in Fig. 2(d,e), where we can see that the amplitude changes due to absorption of radiation, and the phase change occurs. The change of amplitude can be obtained from the conservation of energy

M18 $$\delta \frac{|{{\mathcal{E}}}_{s}{|}^{2}}{4\pi }=\hslash {\omega }_{0}{N}_{0}({n}_{1}-{n}_{2})$$

From Eq. (M17), the field ${{\mathcal{E}}}_{s}$ acquires the phase

M19 $${\Omega }_{s}={\Omega }_{s}^{0}\,\exp \,(i\Theta -\Phi )\simeq {\Omega }_{s}^{0}\,\exp \,(i{\Omega }_{a}^{2}\,{\int }_{-\infty }^{\infty }\,\frac{{\sigma }_{ab}}{|{\Omega }_{s}|}dt),$$

where

$$\Theta ={\Omega }_{a}^{2}\,{\int }_{-\infty }^{\infty }\,\frac{{\rm{Re}}\,[{\sigma }_{ab}]}{|{\Omega }_{s}|}dt,\,{\rm{and}}\,\Phi ={\Omega }_{a}^{2}\,{\int }_{-\infty }^{\infty }\,\frac{{\rm{Im}}\,[{\sigma }_{ab}]}{|{\Omega }_{s}|}dt.$$

For one type of molecule, the estimation of the induced coherence is given by

$$|{\sigma }_{ab}(t)|\simeq \frac{1}{2}\frac{{\Omega }_{s}^{2}}{{\Omega }_{s}^{2}+{({\omega }_{0}-{\omega }_{ab}(t))}^{2}}$$

where the detuning is ${\omega }_{0}-{\omega }_{ab}(t)=\alpha t$, and *α* is the chirping parameter, and the phase is

M20 $$\Theta (t)\simeq \frac{\pi {\Omega }_{a}^{2}}{\alpha }\,\arctan (\frac{\alpha t}{\pi {\Omega }_{0}})$$

The microwave or RF power needed is 5 nW/cm^−2^, 4 mW/cm^−2^, 40 W/cm^−2^ for relaxation rates ${\gamma }_{ph}$: 10^4^ s^−1^, 10^7^ s^−1^, and 10^9^ s^−1^ correspondingly.

### More realistic models: Linear and Symmetrical Molecules

Even though, the physics of the molecules in the electric field is clearly described by the rotator with fixed-axis, for more quantitative estimations, we consider here more realistic models – Linear and Symmetrical Molecules.

The Hamiltonian of the linear molecule with dipole $\overrightarrow{\wp }$ in the dc electric field ${\overrightarrow{{\mathcal{E}}}}_{0}$

M21 $$\hat{H}=\frac{{\hat{J}}^{2}}{2I}-\overrightarrow{\wp }\cdot {\overrightarrow{{\mathcal{E}}}}_{0}$$

For field $${\overrightarrow{{\mathcal{E}}}}_{0}=\overrightarrow{i}{{\mathcal{E}}}_{0}$$,

M22 $$\hat{H}=\frac{{\hat{J}}^{2}}{2I}-\wp {{\mathcal{E}}}_{0}\,\sin \,\theta \,\cos \,\varphi $$

For strong field, the molecule is aligned along $x$ axis, $\theta =\frac{\pi }{2}+\delta \theta$ ($|\delta \theta |\ll 1$, $|\varphi |\ll 1$)

M23 $$\frac{{\hat{J}}^{2}}{2I}=-\,\frac{{\hslash }^{2}}{2I}(\frac{1}{\sin \,\theta }(\frac{\partial }{\partial \theta }\,\sin \,\theta \frac{\partial }{\partial \theta })+\frac{1}{{\sin }^{2}\,\theta }\frac{{\partial }^{2}}{\partial {\varphi }^{2}})\simeq -\,\frac{{\hslash }^{2}}{2I}(\frac{{\partial }^{2}}{\partial {\theta }^{2}}+\frac{{\partial }^{2}}{\partial {\varphi }^{2}})$$

and

M24 $$\begin{array}{rcl}\sin \,\theta \,\cos \,\varphi  & = & \sin (\frac{\pi }{2}+\delta \theta )\,\cos \,\varphi \\  & = & (\sin \,(\frac{\pi }{2})\,\cos \,\delta \theta +\,\cos \,(\frac{\pi }{2})\,\sin (\delta \theta ))\,\cos \,\varphi \end{array}$$

M25 $$\simeq \,\cos \,\delta \theta \,\cos \,\varphi \simeq 1-\frac{\delta {\theta }^{2}}{2}-\frac{{\varphi }^{2}}{2}$$

M26 $$\hat{H}=-\,\frac{{\hslash }^{2}}{2I}(\frac{{\partial }^{2}}{\partial {\theta }^{2}}+\frac{{\partial }^{2}}{\partial {\varphi }^{2}})+\wp {{\mathcal{E}}}_{0}(\frac{\delta {\theta }^{2}}{2}+\frac{{\varphi }^{2}}{2})-\wp {{\mathcal{E}}}_{0}$$

And more generally, for the electric field ${\overrightarrow{{\mathcal{E}}}}_{0}={\overrightarrow{n}}_{0}{{\mathcal{E}}}_{0}$, where ${\overrightarrow{n}}_{0}=\overrightarrow{i}\,\sin \,{\theta }_{0}\,\cos \,{\varphi }_{0}+\overrightarrow{j}\,\sin \,{\theta }_{0}\,\sin \,{\varphi }_{0}+\overrightarrow{k}\,\cos \,{\theta }_{0}$ is the direction of the electric field. Considering close orientation of the molecule $\overrightarrow{n}=\overrightarrow{i}\,\sin \,\theta \,\cos \,\varphi +$ $\overrightarrow{j}\,\sin \,\theta \,\sin \,\varphi +\overrightarrow{k}\,\cos \,\theta$, so $\overrightarrow{n}\cdot {\overrightarrow{n}}_{0}=\,\cos \,\psi$, and we can relate

M27 $${\psi }^{2}={(\theta -{\theta }_{0})}^{2}+{\sin }^{2}\,{\theta }_{0}{(\varphi -{\varphi }_{0})}^{2}$$

introducing ${\psi }_{\theta }=\theta -{\theta }_{0}$ and ${\psi }_{\varphi }=\,\sin \,{\theta }_{0}(\varphi -{\varphi }_{0})$, the Hamiltonian has the form of the one for simple harmonic oscillator and can be written as

M28 $$\hat{H}=-\,\frac{{\hslash }^{2}}{2I}(\frac{{\partial }^{2}}{\partial {\psi }_{\theta }^{2}}+\frac{{\partial }^{2}}{\partial {\psi }_{\varphi }^{2}})+\wp {{\mathcal{E}}}_{0}(\frac{{\psi }_{\theta }^{2}}{2}+\frac{{\psi }_{\varphi }^{2}}{2})-\wp {{\mathcal{E}}}_{0}$$

The Hamiltonian of the symmetrical molecule with dipole $\overrightarrow{\wp }$ in the dc electric field ${\overrightarrow{{\mathcal{E}}}}_{0}$

M29 $$\hat{H}=\frac{{\hat{J}}^{2}}{2{I}_{\perp }}+{\hat{J}}_{\parallel }^{2}(\frac{1}{2{I}_{\parallel }}-\frac{1}{2{I}_{\perp }})-\overrightarrow{\wp }\cdot {\overrightarrow{{\mathcal{E}}}}_{0}$$

**Figure 4:**
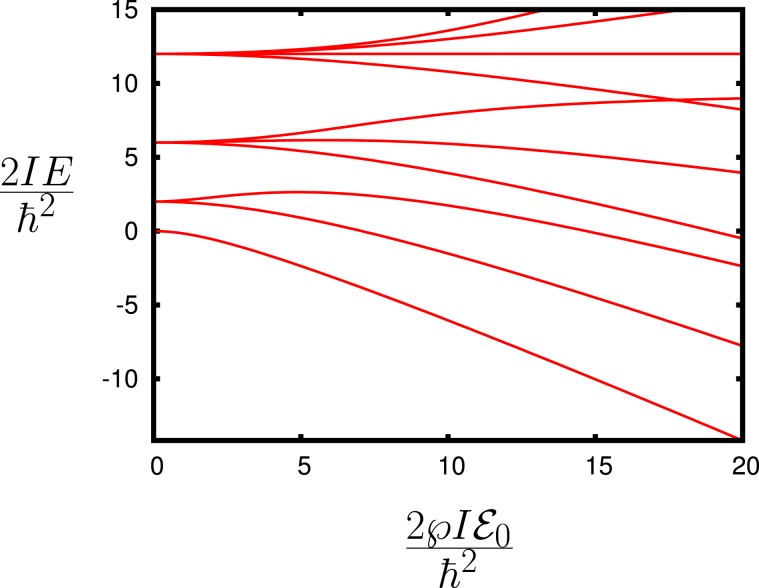
Molecular energy $2I{E}_{JM}/{\hslash }^{2}$ dependence on $2I\wp {{\mathcal{E}}}_{0}/{\hslash }^{2}$.

**Figure 5:**
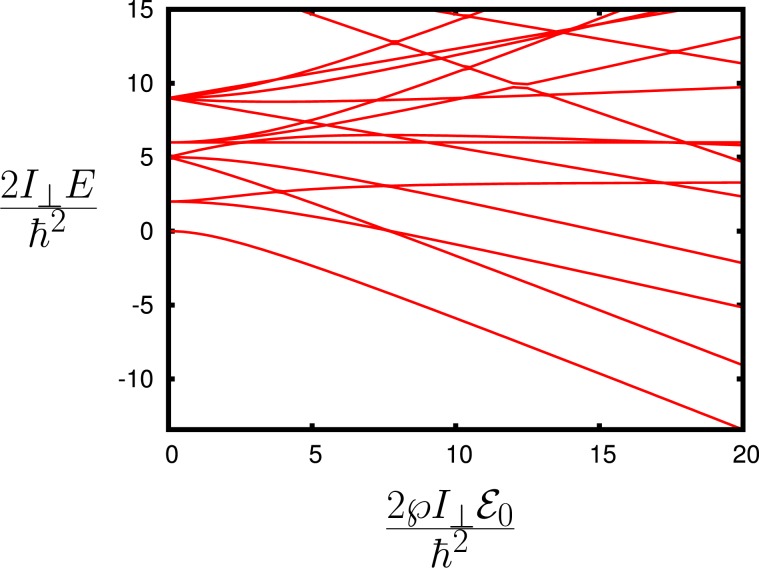
Molecular energy $2{I}_{\perp }{E}_{JMk}/{\hslash }^{2}$ dependence on $2{I}_{\perp }\wp {{\mathcal{E}}}_{0}/{\hslash }^{2}$.

The functions ${D}_{Mk}^{(J)}(\alpha ,\beta ,\gamma )$ are the eigenfunctions of the Hamiltonian without the electric field ${{\mathcal{E}}}_{0}$ and $\overrightarrow{\wp }\cdot {\overrightarrow{{\mathcal{E}}}}_{0}=\wp {{\mathcal{E}}}_{0}\,\cos \,\beta$. The energy levels are shown in Figs. 4 and 5.
